# 能谱CT定量参数：术前诊断肺癌转移性淋巴结的价值

**DOI:** 10.3779/j.issn.1009-3419.2016.11.04

**Published:** 2016-11-20

**Authors:** 峰峰 杨, 杰 董, 晓龙 闫, 秀婷 王, 小娇 付, 同 张

**Affiliations:** 1 150001 哈尔滨，哈尔滨医科大学附属第四医院放射科 Department of Radiology, the Fourth Affiliated Hospital, Harbin Medical University, Harbin 150001, China; 2 150001 哈尔滨，哈尔滨医科大学附属第一医院干部病房 Department of Geriatrics, the First Affiliated Hospital, Harbin Medical University, Harbin 150001, China

**Keywords:** 能谱CT, 肺肿瘤, 淋巴结转移, Gemstone spectral CT, Lung neoplasms, Lymphnode metastasis

## Abstract

**背景与目的:**

肺癌淋巴结转移是重要的生存预后因素，准确的纵隔淋巴结分期可以使患者最大程度地受益于手术。本研究旨在探讨宝石能谱计算机断层扫描（computed tomography, CT）定量参数在术前诊断非小细胞肺癌（non-small cell lung cancer, NSCLC）患者淋巴结转移的价值。

**方法:**

收集48例NSCLC患者，连续进行宝石能谱CT成像（gemstone spectral imaging, GSI）模式肺增强扫描和手术治疗。重建GSI数据，测量淋巴结的大小、动脉期和静脉期的CT值、能谱曲线的斜率、标准化碘浓度以及水浓度。采用独立样本的*t*检验，并进行受试者工作特征曲线（receiver operating characteristic, ROC）分析，确定诊断的最佳阈值及效能。

**结果:**

转移性淋巴结与非转移性淋巴结短轴直径、动脉期和静脉期的CT值、能谱曲线的斜率、标准化碘浓度均有统计学差异。当确定动脉期能谱曲线斜率的最佳临界值为2.75，其诊断的敏感性、特异性及总体的准确性分别为88.2%、88.4%、87.0%。

**结论:**

能谱CT的GSI模式定量参数较传统CT在术前诊断转移性淋巴结方面有更高的诊断效能。

## Abstract (en)

**Background and objective:**

Mediastinal involvement in lung cancer is an important prognostic factor affecting survival, and accurate staging of the mediastinum lymph node correctly identifies patients who can benefit the most from surgery. The aim of this study is to investigate the value of dual-energy spectral computed tomography (DEsCT) imaging in differentiating metastatic from non-metastatic lymph nodes in lung cancer.

**Methods:**

Forty-eight patients with non-small cell lung cancer (NSCLC) underwent arterial (AP) and portal venous (PP) phase contrast-enhanced DEsCT imaging followed by surgical treatment. gemstone spectral imaging (GSI) data images were reconstructed and transmitted to an offline workstation. GSI quantitative parameters, including lymph-node size, CT value, IC, water concentration, and spectral curve. Differences were tested for statistical significance using the two-sample *t* test. ROC analysis was performed to assess diagnostic performance.

**Results:**

The mean short-axis diameter of metastatic LNs, slope of the spectral Hounsfield unit curve (λHU), normalized iodine concentration measured during, and both AP and PP were significantly higher in metastatic lymph node than that in benign lymph nodes. The best parameter for detecting metastatic lymph nodes was AP λHU when a threshold λHU of 2.75 was used; sensitivity, specificity, and accuracy were 88.2%, 88.4%, and 87.0%, respectively.

**Conclusion:**

Quantitative assessment with gemstone spectral imaging quantitative parameters showed higher accuracy than the qualitative assessment of conventional CT imaging features for the preoperative diagnosis of metastatic lymph nodes in patients with lung cancer.

肺癌是恶性肿瘤的头号杀手^[1]^，尽管在治疗方面有一定进展，但是其预后仍相对较差^[1, 2]^。肺癌的准确分期与治疗和预后密切相关，特别是纵隔淋巴结的分期^[3]^。传统CT在诊断肺癌淋巴结转移的准确率约为60%^[4-6]^。当前的正电子发射体层显像/计算机体层成像（positron emission tomography/computed tomography, PET/CT）空间分辨率也妨碍了对小淋巴结转移灶的检测^[6]^。

胸腔镜手术和支气管内超声等侵入性的评估手段，可以明确诊断，但可引起严重的并发症^[7-10]^。此外，由于淋巴结位置的特殊性，大大增加检测的难度。因此，需要一种能更准确的表征纵隔淋巴结或确定是否需要和选择最佳有创分期手段的非侵入性检查方法。

宝石能谱CT成像（gemstone spectral imaging, GSI）模式是在一次旋转中通过完成80 KVp和140 KVp瞬时切换来采集数据。与传统的混合能量CT相比，能谱CT可以产生从40 KeV到140 KeV条件下的单能量图像、物质分离图像、有效原子序数和能谱曲线等定量参数，从而放大不同组织来源的细微差别^[11-13]^。能谱CT的定量参数在临床多用于诊断肺栓塞^[14]^、不同类型肿瘤的诊断^[15-17]^和胃癌患者淋巴结分期^[18]^。我们研究的目的是探讨宝石能谱CT定量参数在术前鉴别诊断非小细胞肺癌（non-small cell lung cancer, NSCLC）患者转移性淋巴结的应用价值。

## 1 材料与方法

### 1.1 研究对象

项前瞻性研究经由我们机构的审查委员会批准，征得患者的知情同意，并告知相应的注意事项。收集2014年12月-2015年12月来我院就诊怀疑肺占位的患者，予以胸部GSI模式双期增强扫描，并需满足下列条件者被纳入本研究。纳入标准：①没有对胸部肿瘤进行过治疗（如放射治疗或化学治疗、穿刺活检等）；②CT横轴位淋巴结的短轴直径≥5 mm；③所有的良性与转移性淋巴结均经病理证实；④所有的转移性淋巴结，病理诊断均为NSCLC转移；⑤图像为能谱模式，清晰，无明显运动伪影，可进一步分析。本研究中共有64例患者接受胸部GSI模式扫描，48例患者接受手术切除和淋巴结清扫术，其中男性29例，女性19例，平均年龄（60.7±8.9）岁。肺癌病理类型：腺癌24例，鳞状细胞癌21例，不典型类癌3例。因16例缺少病理结果被排除。详细的实验流程图见图 1。

**1:**
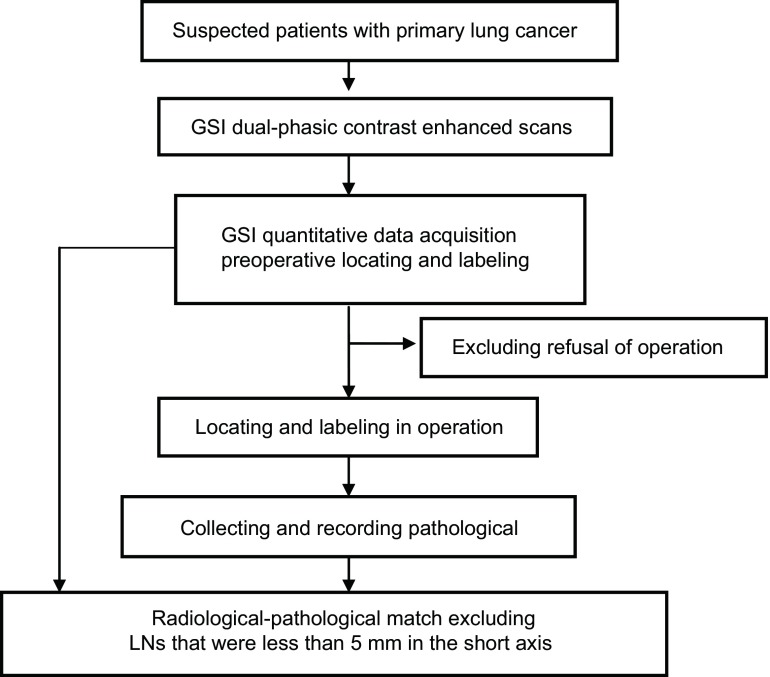
实验流程图 Strategy for lymph node analysis and data processing in this study

### 1.2 GSI成像方法

采用第二代宝石能谱CT（Discovery CT 750 HD, GE Healthcare, USA）对所有的患者使用GSI模式进行肺双期增强扫描。扫描范围起自胸廓入口处到肺底水平，保证完全覆盖肺组织。使用双筒高压注射器以3.5 mL/s经肘静脉注入非离子型碘对比剂碘佛醇（Ioverysol, 350 mgI/mL）70 mL，然后分别经35 s、90 s后进入动脉期和静脉期。GSI模式其他参数如下：机架旋转时间0.6 s，螺距1.375，管电流600 mA，该模式下相对应的容积剂量指数是18.28 mGy。重建的GSI数据图像的层厚和层间隔均为1.25 mm，然后传输到附带GSI viewer软件包的后处理工作站（GE Advantage Workstation AW4.5, GE Healthcare）进行图像分析。

### 1.3 图像定量参数的采集

由两名分别具有10年以上工作经验的胸部放射科医师在GE AW4.5（GE Healthcare, USA）后处理工作站且在不告知病理结果的情况下独立的完成定量参数的采集，当有分歧时，经过协商达成一致。在重建的70 KeV图像中，选取目标淋巴结的最大层面放置感兴趣区，感兴趣区的面积要尽可能的包括淋巴结同时尽量避开淋巴结周围脂肪组织、坏死及钙化区。所有的感兴趣区均重复测量三次，取其平均值。

在70 KeV图像中测量目标淋巴结横轴位图像上的最短直径。GSI viewer软件可以在碘基物质分离图像和水基物质分离图像中自动计算感兴趣区内的碘浓度和水浓度。为了减小可能由于患者循环状况或扫描时间导致的差异，每一个感兴趣区内的碘浓度（iodine concentration, IC）除以同层的胸主动脉的碘浓度计算得出标准化的碘浓度（normalized iodine concentration, NIC）：NIC=IC/ICaorta。GSI viewer软件还可以自动计算所有单能量图像的CT值，对每一个感兴趣区形成一条能谱衰减曲线。分别计算目标淋巴结的能谱衰减曲线的斜率（λHU）：λHU=（CT_40 keV_-CT_100 keV_）/60。

### 1.4 淋巴结一对一的匹配

为更有助于对淋巴结进行放射学与病理学一对一的比较，遵循国际肺癌研究联合会修订的第七版中N分期的标准对入组的淋巴结进行准确分区^[19]^。在术中对清扫的淋巴结进行详细的描述（如：位置、大小、距离等）与标记，记录后送检。根据获得的病理结果，如果某一组中淋巴结均是转移淋巴结，那么相应的CT图像上该对应组的所有的淋巴结被纳入转移性淋巴结组。同样的方法适用于非转移性淋巴结。如果病理回报一组淋巴结中既有转移性淋巴结同时又包括非转移性淋巴结，那么我们需要借助术中对淋巴结所做的标记和详细的描述来追踪该区域的淋巴^[20]^，尽可能完成淋巴结一对一的匹配。

### 1.5 统计学方法

所有的定量数据均采用SPSS 22.0软件包进行数据分析，计量资料均用均数±标准差表示。所有的定量参数均进行独立样本的*t*检验，绘制接受者操作特征曲线（receiver operating characteristic, ROC），通过最高*Youden’s J*统计值确定被测变量的最佳临界值。计算ROC曲线下的面积（area under the receiver operating characteristic curve, AUC）来分析对比各种参数的诊断效能。当取最佳临界值时，计算各个能谱CT定量参数诊断的敏感性、特异性、阳性预测值、阴性预测值，取相应的95%CI。以*P* < 0.05为差异有统计学意义。

## 2 结果

### 2.1 患者和淋巴结

48例NSCLC患者经病理证实的淋巴结共计198枚，按照上述淋巴结一对一匹配的标准进行标记，其中90枚淋巴结被排除，由于淋巴结的位置比较特殊难以准确定位。最终确定有效的淋巴结共计108枚，按淋巴结是否发生转移对其进行分组，其中转移性淋巴结组48枚，而非转移性淋巴结组60枚。本研究中淋巴结在各分区的分布情况见表 1。

**1:** 108个淋巴结的分布 Distribution of all 108 lymph nodes

Levels	Benign lymph nodes (*n*=60)	Metastatic lymph nodes (*n*=48)
1	0	0
2	5	4
3	4	7
4	13	12
5	11	9
6	7	5
7	15	9
8	0	0
9	0	0
10	5	2
Data are number of lymph nodes; Level means station of lymph nodes.		

### 2.2 转移性淋巴结与非转移性淋巴结的定量参数分析

#### 2.2.1 淋巴结的大小

在70 Kev单能量图像中，测得所有转移性淋巴结的短轴直径为（1.13±0.24）mm，而非转移性淋巴结为（0.77±0.24）mm。转移性淋巴结短轴直径的平均值比非转移组淋巴结增大（*P* < 0.05）。仅58.3%（63/108）的淋巴结大于或等于10 mm，其中≥10 mm的转移性淋巴结40枚（37.0%, 40/108），此外，小于10 mm的转移性淋巴结8枚（7.4%, 8/108）。本研究中按既定的淋巴结最大短轴直径≥10 mm为诊断标准，其诊断淋巴结转移的敏感性为83.3%，特异性为61.7%，准确性为71.3%（表 2）。

**2:** 常规CT形态学与病理学结果的比较 Comparison CT morphologic diagnosis of lymph node metastasis with pathologic diagnosis

Pathological diagnosis groups	Conventional CT features		Total
	≥10 mm	< 10 mm	
Benign lymph nodes	23 (38.3%)	37 (61.7%)	60 (100.0%)
Metastatic lymph nodes	40 (83.3%)	8 (16.7%)	48 (100.0%)
Total	63	45	108
CT: computed tomography.			

#### 2.2.2 淋巴结的CT值、能谱曲线斜率、标准化碘浓度和水浓度

转移性淋巴结和非转移性淋巴结在动脉期和静脉期的CT值、能谱曲线的斜率、标准化碘浓度均有统计学差异（*P* < 0.05）（图 2和图 3）。而转移性淋巴结和非转移性淋巴结在水基物质分离图像中获得的动脉期和静脉期的水浓度均无统计学差异（*P*值分别为0.105、0.800）（表 3）。

**2:**
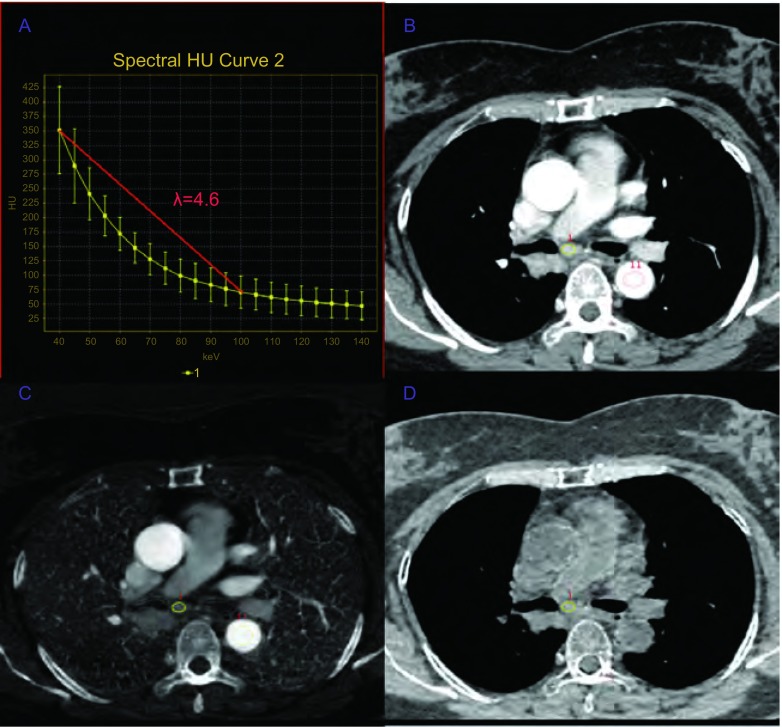
患者女性，52岁，术后病理为右主支气管腺癌，临床分期T1N2M0，第7组淋巴结为转移性淋巴结。A：第7组淋巴结动脉期的能谱曲线斜率为4.6，提示该淋巴结为转移性的；B：70 keV单能量增强图像动脉期显示7组淋巴结肿大。短轴直径为9 mm，CT值为124.1 HU；C：该淋巴结动脉期碘基物质分离图像的碘浓度为3.82 mg/cm^3^，同层胸主动脉的碘浓度为15.43 mg/cm^3^，该淋巴结的标准化碘浓度为0.25；D：该淋巴结动脉期水基物质分离图像的水浓度为1, 024.3 mg/cm^3^。 A 52-year-old female patient, postoperative pathological diagnosis was right main bronchus adenocarcinoma, clinical stage was T1N2M0, seventh group of lymph nodes were metastatic lymph nodes. A: λHU of seventh group in arterial phase was 4.6, indicating this lymph node was metastatic; B: 70 keV monochromatic image in arterial phase shows that seventh group of lymph nodes expand. Size in the axis image was 9 mm. CT value, 124.1 HU; C: Iodine-based material-decomposition image shows that ICs in the lymph node was 3.82 mg/cm^3^ and ICaorta of thoracic aorta in the same slice was 15.43 mg/cm^3^, NIC of the lymph node was 0.25; D: Water-based material-decomposition image shows that water concentration in the lymph node was 1, 024.3 mg/cm^3^.

**3:**
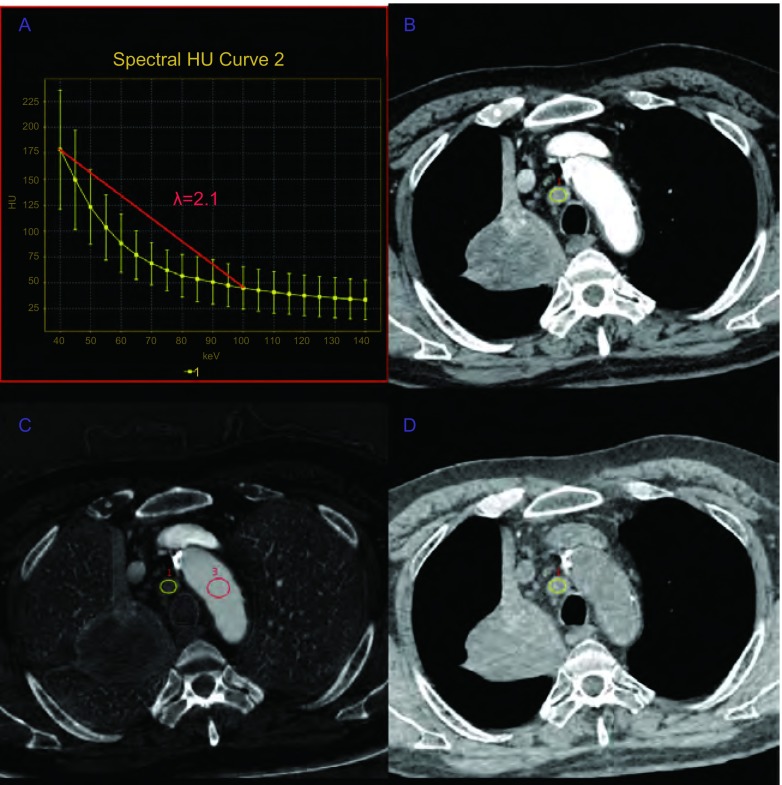
患者男性，59岁，术后病理为右肺上叶高分化鳞癌，临床分期T2N0M0，第4组淋巴结未见转移。A：第4组淋巴结动脉期的能谱曲线斜率为2.1，提示该淋巴结为非转移性的；B：70 keV单能量增强图像动脉期显示4组淋巴结肿大。短轴直径为12 mm，CT值为62.1 HU；C：该淋巴结动脉期碘基物质分离图像的碘浓度为1.64 mg/cm^3^，同层胸主动脉的碘浓度为8.83 mg/cm^3^，该淋巴结的标准化碘浓度为0.19；D：该淋巴结动脉期水基物质分离图像的水浓度为1, 024.2 mg/cm^3^。 A 59-year-old male patient, postoperative pathological diagnosis was right upper lobe well-differentiated squamous cell carcinoma, clinical stage was T2N0M0, fourth group of lymph nodes were non-metastatic lymph nodes. A: λHU of fourth group in arterial phase was 2.1, indicating this lymph node was non-metastatic; B: 70 keV monochromatic image in arterial phase shows that fourth group of lymph nodes expand. Size in the axis image was 12 mm. CT value, 62.1 HU; C: Iodine-based material-decomposition image shows that ICs in the lymph node was 1.64 mg/cm^3^ and ICaorta of thoracic aorta in the same slice was 8.83 mg/cm^3^, NIC of the lymph node was 0.19; D: Water-based material-decomposition image shows that water concentration in the lymph node was 1, 024.2 mg/cm^3^.

**3:** 转移性淋巴结与肺转移性淋巴结GSI定量参数的比较 Difference of GSI quantitative parameters between benign and metastatic lymph nodes

Parameter	Benign lymph nodes	Metastatic lymph nodes	*P* value
Size	0.77±0.24	1.13±0.24	< 0.001
AP CT value	70.0±17.4	106.2±19.1	< 0.001
AP *λ*HU	2.1±0.5	3.6±0.9	< 0.001
AP NIC	0.16±0.05	0.22±0.05	< 0.001
AP WC	1, 024.9±15.5	1, 030.1±10.8	0.105
VP CT value	69.5±16.5	85.5±15.9	< 0.001
VP λHU	2.3±0.6	2.7±0.8	0.005
VP NIC	0.49±0.12	0.57±0.14	0.009
VP WC	1, 022.1±14.7	1, 022.8±11.3	0.800
AP: arterial phase; VP: venous phase; *λ*HU: The slope of the Hounsfieldunit curve; NIC: normalized iodine concentration values; WC: water concentration values；GSI: gemstone spectral imaging; data are mean values±standard deviations; *P* < 0.05 indicates a statistically significant difference.			

#### 2.2.3 接受者操作特征曲线ROC分析

绘制淋巴结的大小，动脉期和静脉期的CT值、能谱曲线的斜率、标准化碘浓度的ROC曲线，确定每个定量参数的最佳临界值（图 4）。所有的ROC曲线均在参考线之上。鉴别诊断转移性淋巴结和非转移性淋巴结的各个定量参数的曲线下面积、最佳临界值、敏感性、特异性、阳性预测值、阴性预测值见表 4。与其他的定量参数相比，动脉期能谱曲线的斜率的曲线下面积（area under curve, AUC=0.951）最大。如果确定动脉期的能谱曲线斜率的最佳临界值为2.75，那么其诊断的敏感性、特异性、阳性预测值、阴性预测值及总体的准确性分别为88.2%、88.4%、85.8%、90.4%、87.0%。

**4:**
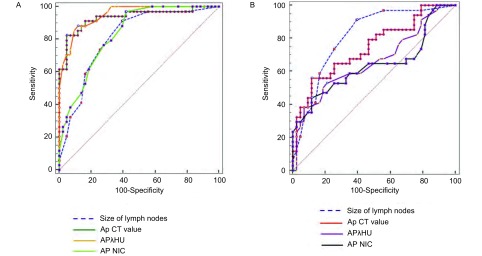
转移性淋巴结与非转移性淋巴结GSI定量参数的ROC曲线分析。A：动脉期能谱CT定量参数诊断NSCLC患者转移性淋巴结与非转移性淋巴结的ROC曲线；B：静脉期能谱CT定量参数诊断NSCLC患者转移性淋巴结与非转移性淋巴结的ROC曲线。所有的ROC曲线均在参考线之上。 Graphs show receiver operating characteristic curves of GSI quantitative parameters of benign and metastatic lymph nodes in patients with NSCLC. A: Receiver operating characteristic curves of GSI quantitative parameters in arterial phase for differentiating benign and metastatic lymph nodes in patients with NSCLC; B: Receiver operating characteristic curves of GSI quantitative parameters in venous phase of benign and metastatic lymph nodes in patients with NSCLC. All ROC curves are above the reference line. ROC: receiver operating characteristic curve.

**4:** 转移性淋巴结与非转移性淋巴结GSI定量参数的诊断效能 GSI quantitative parameters for differential diagnosis of benign and metastatic lymph nodes in patients with NSCLC GSI

Quantitative parameters	AUC	Threshold	Sensitivity (%)	Specificity (%)	PPV (%)	NPV (%)
Size of lymph node	0.780	0.7	90.6 (75.0-98.0)	56.3 (37.7-73.6)	87.1 (74.7-94.8)	64.8 (34.6-88.2)
AP CT value	0.934	92.1	82.4 (65.5-93.2)	95.3 (84.2-99.4)	93.3 (77.9-99.2)	87.2 (74.3-95.2)
AP *λ*HU	0.951	2.75	88.2 (72.5-96.7)	88.4 (74.9-96.1)	85.8 (69.8-95.2)	90.4 (77.3-97.3)
AP NIC	0.833	0.15	97.1 (84.7-99.9)	58.1 (42.1-73.0)	64.7 (50.1-77.6)	96.2 (80.4-99.9)
VP CT value	0.744	83.0	55.9 (37.9-72.8)	88.4 (74.9-96.1)	79.2 (57.9-92.9)	71.7 (57.6-83.2)
VP λHU	0.662	2.5	52.9 (35.1-70.2)	79.1 (64.0-90.0)	66.7 (46.1-83.5)	68.0 (53.3-80.4)
VP NIC	0.638	0.6	44.1 (27.2-62.1)	88.4 (74.9-96.1)	75.1 (51.0-91.4)	66.6 (52.9-78.6)
AUC: area under the receiver operating characteristic curve; NSCLC: non-small cell lung cancer; Data in parentheses are 95%CIs.						

## 3 讨论

淋巴结的分期是影响肺癌患者治疗和预后的重要因素，正如其他肿瘤一样，由于微转移或炎性病变均会导致淋巴结的肿大，因此，检测淋巴结转移仍是一个具有挑战性的问题^[20]^。如何准确地评价肺癌患者淋巴结受累情况，一直备受临床医生的关注。传统的CT诊断根据既定的大小的标准（淋巴结短轴直径≥10 mm）作为判断淋巴结转移的依据，传统的CT在诊断恶性肿瘤淋巴结转移方面的敏感性和特异性分别为51%和85%^[21]^。在我们的研究中也提示该诊断标准存在一定的局限性，因为存在短轴直径 < 10 mm的淋巴结病理证实已经发生了转移，然而有些短轴直径≥10 mm的淋巴结却并未发生转移，而是因为炎性反应性增生致使淋巴结肿大。仅考虑淋巴结短轴直径这一单一因素，在本研究中，当淋巴结短轴直径的最佳阈值定位7 mm时，此时ROC AUC为0.780，敏感性和特异性分别为90.6%、56.3%。在目前的研究中，我们发现GSI定量参数中，尤其动脉期能谱曲线的斜率的AUC=0.951是最大的。如果确定动脉期的能谱曲线斜率的最佳临界值为2.75，那么其诊断的敏感性、特异性、阳性预测值、阴性预测值及总体的准确性分别为88.2%、88.4%、85.8%、90.4%、87.0%。与传统CT的标准对转移性淋巴结的诊断相比，在术前诊断NSCLC患者淋巴结转移有更高的精准度。

能谱CT成像以瞬时双KVp切换为特性，与常规CT图像相比，能够有效地规避射线的硬化效应，在每一个单能量水平都可以得到一个对应的精确的CT值，从而形成了CT值随KeV值变化而变化的能谱衰减曲线。不同能量水平的CT值反映了淋巴结在不同能量水平下的质量吸收系数，而能谱衰减曲线则反映了质量吸收系数随能量变化而变化的关系。Li等^[22]^的研究表明不同化学构成的组织具有不同的能谱衰减曲线，因此可以用能谱衰减曲线来区分不同化学组成的病灶。在我们的研究中，转移性淋巴结在动脉期的能谱曲线的斜率与非转移性淋巴结明显不同，体现出转移性淋巴结与非转移性淋巴结的非同源性。

此外，能谱CT成像还可以产生基于碘和水的物质分离图像，可用于精准定量碘浓度的测量^[12]^。原则上物质对的选择是没有局限的，可以是自然界的任意两种物质，就医学的成像诊断而言，碘和水是最常用的物质对，因为它们的原子序数跨度较大，因此形成的衰减差异较大，这种对比就形成了能直观解释的物质衰减图像^[14, 15]^。能谱CT的定量参数在临床已用于肺癌^[23]^和不同类型肿瘤的鉴别诊断^[15-17]^。在本研究中，转移性淋巴结在动脉期及静脉期的标准化碘浓度均高于非转移性淋巴结，与Liu等^[24]^的研究相符。这种差异考虑主要为转移性淋巴结内微血管床数目的增加导致瘤体的灌注增加所致。

[^18^F]-葡萄糖正电子发射计算机断层（fluorodeoxyglucose positron emission tomography/computed tomograohy, FDG-PET/CT）扫描可以评估淋巴结的代谢情况，被广泛用于肺癌患者的淋巴结分期。总体而言，PET/CT扫描识别转移性淋巴结的敏感性和特异性分别为约77%和86%^[6]^。虽然我们的研究中，没有进行能谱CT的定量参数和PET/CT的比较，但是我们的研究结果显示，动脉期能谱曲线的斜率，与报道的PET/CT对转移性淋巴结的诊断性能相比，在术前诊断NSCLC患者淋巴结转移有更高的精准度。但是，进一步的研究应该是关于具体比较能谱CT的GSI模式成像和PET/CT扫描在术前诊断肺癌转移性淋巴结的价值，这是非常有必要的。

我们研究中仍存在一些不足需要考虑：①这是我们的初步研究结果，还有待大样本量的研究加以验证。在本研究中小于5 mm的淋巴结被排除，尽管我们认真执行淋巴结一对一匹配的标准，但是仍可能造成不完全匹配；②因为它不是我们的主要目的，所以本研究中对非转移性淋巴结的组织病理学类型没有具体区分，其中包含正常淋巴结和炎性反应性增生性淋巴结；③考虑到现有的能谱CT的GSI模式成像的辐射剂量仍然较高，随着更低剂量GSI扫描技术的出现及推广应用，其临床的应用可能会更加的广泛。

综上所述，宝石能谱CT的GSI模式成像的定量参数较传统CT在术前鉴别诊断转移性淋巴结和非转移性淋巴结方面有更高的诊断效能。
